# Dog temperament is correlated with body weight and climate in country of origin

**DOI:** 10.1093/cz/zoaf044

**Published:** 2025-08-01

**Authors:** Ai Matsumoto, James A Serpell, Miho Nagasawa, Takefumi Kikusui

**Affiliations:** School of Veterinary Medicine, Azabu University, 1-17-71 Fuchinobe, Chuo-ku, Sagamihara, Kanagawa 252-5201, Japan; School of Veterinary Medicine, University of Pennsylvania, Philadelphia, PA 19104, USA; School of Veterinary Medicine, Azabu University, 1-17-71 Fuchinobe, Chuo-ku, Sagamihara, Kanagawa 252-5201, Japan; School of Veterinary Medicine, Azabu University, 1-17-71 Fuchinobe, Chuo-ku, Sagamihara, Kanagawa 252-5201, Japan

**Keywords:** aggression, breed, physiological parameter, temperature

Among all animals, dogs were domesticated the earliest and formed a unique bond with humans. Hare and Tomasello proposed that in ancestral canids, selection for individuals with reduced fear and aggression allowed them to coexist in a niche adjacent to the human niche (Hare and Tomasello 2005). As a byproduct of this process, dogs acquired social cognitive abilities that facilitated communication with humans. Subsequently, through artificial selection, further beneficial traits were favored, resulting in a wide variety of forms, abilities, and remarkable adaptability to human society that we see today.

Dogs have accompanied humans, and spread worldwide during the early stages of domestication. Adaptations to various aspects of human lifestyle and the environment have led to the acquisition of diverse characteristics. Physiologically, dogs have adapted to consume human leftovers by enhancing starch digestion via changes in the copy number of amylase-encoding genes (Axelsson et al. 2013). Additionally, behavioral adaptations have been selected to integrate into human society, with several genetic mutations known to influence dog behavior. However, the mechanism by which genetic polymorphisms associated with dog temperament arose and was subsequently selected for in breed creation, as proposed by the emotional reactivity hypothesis (Hare and Tomasello 2005), is not clear. This process may have been influenced by both natural adaptation to the environment and artificial selection.

Previous studies have shown that human personality traits vary by geographic region, with Wei et al. (2017) highlighting the significance of temperature as a factor in shaping personality traits. This study found that humans are more extraverted and open-minded in warmer environments. This effect of climate on temperament may also be seen in dogs, which have lived in proximity to humans for a long time. For example, dogs living with extraverted humans in warmer temperature regions may have decreased aggression and fear of strange humans and other dogs. Alternatively, dogs that were not suited to environments with high human interaction may have adapted by moving away from such communities. In animal models, hormones involved in metabolism alter social behavior. For instance, cortisol is a glucose-metabolizing hormone that increases the body's temperature expenditure but also acts centrally to increase aggression and activity. Oxytocin increases thermogenesis and suppresses appetite but centrally regulates interindividual relationships and attachment.

We hypothesize that local climate and associated human societies played a role in the genetic selection of dog temperaments during breed creation. This study aimed to explore the relationships among breed-specific temperament in dogs, temperature in countries of origin, and traits of human society.

The Canine Behavioral Assessment and Research Questionnaire (C-BARQ) is a validated tool for assessing a wide range of canine behavioral traits based on owner-reported data (Duffy and Serpell 2012; Hsu and Serpell 2003; Table 1). Correlation analysis of the C-BARQ scores showed that temperature in the country of origin and dog weight was primarily associated with temperament. The higher the temperature in the country of origin, the higher the breed-specific aggression of dogs toward strange humans and other dogs (Pearson’s *r* = 0.346, *P* < 0.001, Figure 1A; *r* = 0.326, *P* < 0.001, Figure 1B) and even greater aggression toward their dog housemates (*r* = 0.219, *P* < 0.01, Supplementary Figure S1b). Higher temperatures were also associated with higher breed-specific fear of strange humans, strange dogs, and nonsocial stimuli (*r* = 0.496, *P* < 0.001, Figure 1C; *r* = 0.365, *P* < 0.001, Figure 1D; *r* = 0.291, *P* < 0.001, Supplementary Figure S1c), as well as heightened sensitivity to potentially painful procedures (*r* = 0.303, *P* < 0.001, Supplementary Figure S1g). Furthermore, the higher the temperature, the more the dog breeds sought attachment and attention and exhibited more separation-related problems (*r* = 0.255, *P* < 0.01, Figure 1F; *r* = 0.205, *P* < 0.05, Figure 1E).

**Figure 1.**
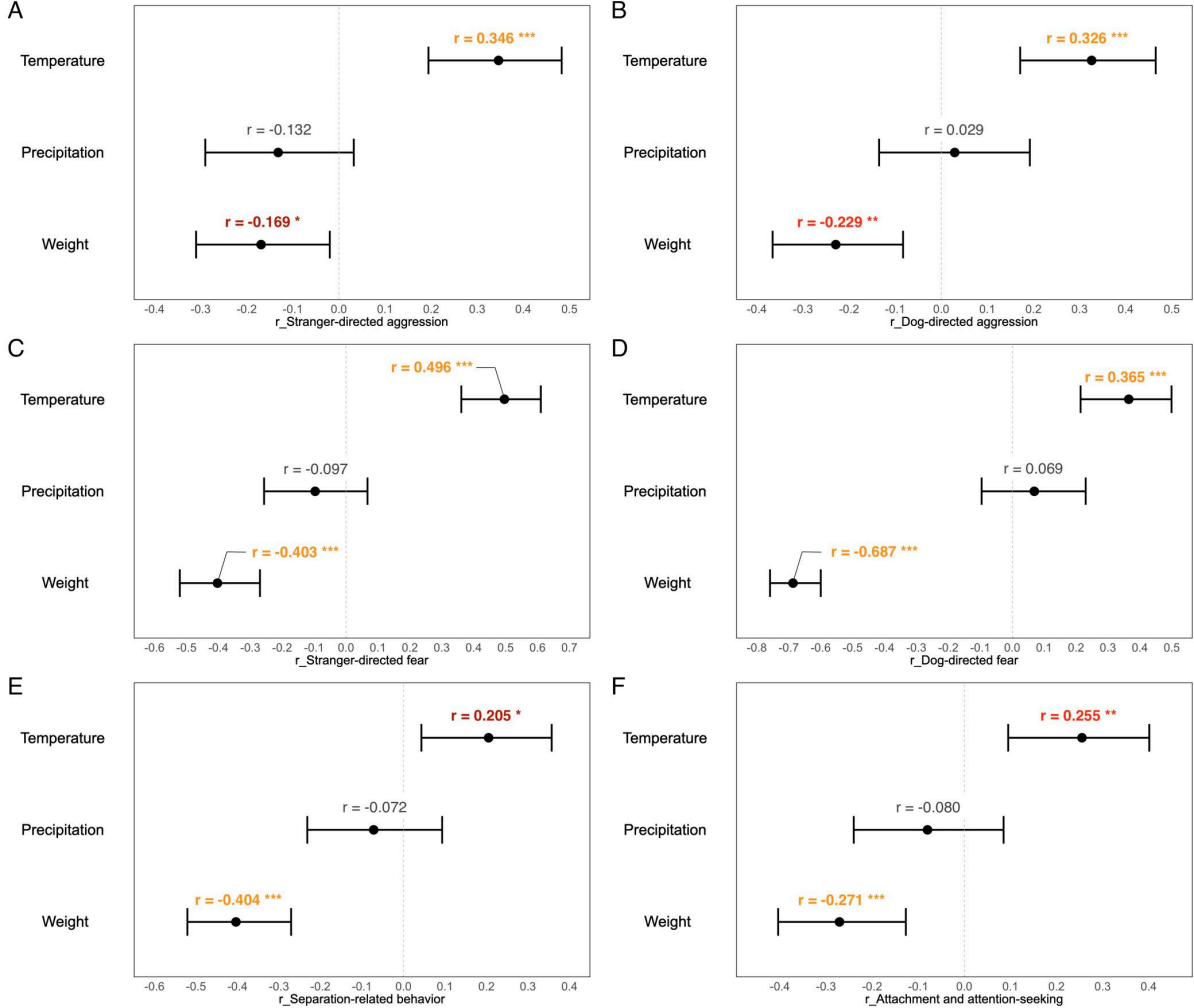
Forest plots showing the correlations between the average annual temperature, annual precipitation, breed-average body weight, and behavioral trait scores from the C-BARQ. Panels (A–F) correspond to different behavioral traits: (A) Stranger-directed aggression, (B) Dog-directed aggression, (C) Stranger-directed fear, (D) Dog-directed fear, (E) Separation-related behavior, and (F) Attachment and attention-seeking. Dots indicate Pearson’s correlation coefficients (*r*), and horizontal bars represent the 95% confidence intervals. Asterisks denote statistical significance (*P < 0.05, **P < 0.01, ***P < 0.001).

**Table 1.** Descriptions of behavioral categories assessed by the C-BARQ.

C-BARQ category	Description
Stranger-directed aggression	Aggressive or threatening behaviors, such as growling, barking, or snapping, displayed toward unfamiliar people who approach or intrude on the dog's or owner's personal space, territory, or home environment.
Owner-directed aggression	Hostile or threatening behaviors shown toward the owner or other household members in situations involving physical manipulation, direct eye contact, stepping over the dog, or approaching whereas the dog possesses food or valued objects.
Dog-directed aggression	Aggressive responses, including growling or snapping, exhibited when encountering unfamiliar dogs.
Dog rivalry	Aggressive or hostile behaviors displayed toward other familiar dogs living in the same household.
Stranger-directed fear	Fearful, anxious, or wary reactions when approached or confronted by unfamiliar people.
Nonsocial fear	Fearful or cautious responses triggered by nonsocial stimuli such as sudden loud noises, moving vehicles, unfamiliar objects, or new situations.
Dog-directed fear	Fearful, wary, or avoidant behaviors shown in response to unfamiliar dogs.
Separation-related behavior	Behaviors such as vocalization, destructiveness, or signs of anxiety (e.g., restlessness, loss of appetite, trembling, excessive salivation) occurring when separated from the owner.
Attachment and attention-seeking	Persistent behaviors aimed at maintaining proximity to the owner or household members, seeking physical affection or attention, and displaying agitation when the owner interacts with others.
Trainability	The dog's responsiveness to the owner, including willingness to obey basic commands, learn new tasks quickly, retrieve objects, respond positively to corrections, and remain focused despite distractions.
Chasing	The tendency to pursue cats, birds, or other small animals when given the opportunity.
Excitability	Intense and prolonged arousal in response to exciting events such as walks, car rides, doorbells, visitor arrivals, or the owner's return home, often accompanied by difficulty calming down afterward.
Touch sensitivity	Fearful or anxious responses to procedures involving physical handling, such as grooming, bathing, nail trimming, or veterinary examinations.
Energy level	High levels of general activity and playfulness, characterized by frequent movement and energetic behavior.

Behavioral categories are listed as defined in the original C-BARQ instrument (Duffy and Serpell 2012; Hsu and Serpell 2003).

Dog breeds with lower body weight exhibited higher aggression toward strange humans, strange dogs, their owners, and their dog housemates (*r* = −0.169, *P* < 0.05, Figure 1A; *r* = −0.229, *P* < 0.01, Figure 1B; *r* = −0.415, *P* < 0.001, Supplementary Figure S1a; *r* = −0.368, *P* < 0.001, Supplementary Figure S1b). Lower weight was also linked to greater fear of strange humans, strange dogs, and nonsocial stimuli (*r* = −0.403, *P* < 0.001, Figure 1C; *r* = −0.687, *P* < 0.001, Figure 1D; *r* = −0.456, *P* < 0.001, Supplementary Figure S1c). Additionally, dog breeds with lower weight were also more sensitive to potentially painful procedures (*r* = −0.612, *P* < 0.001, Supplementary Figure S1g) and were more likely to seek attachment and attention (*r* = −0.271, *P* < 0.001, Figure 1F), as well as to experience separation-related problems (*r* = −0.404, *P* < 0.001, Figure 1E). Furthermore, dog breeds with lower weight were more prone to excitement and had higher energy levels (*r* = −0.405, *P* < 0.001, Supplementary Figure S1f; *r* = −0.211, *P* < 0.01, Supplementary Figure S1h).

Precipitation in the country of origin was not significantly correlated with temperament categories.

Temperature–precipitation and precipitation–weight were not significantly correlated (*P* = 0.196; *P* = 0.831, respectively); whereas, there was a significant negative correlation for temperature-weight (*r* = −0.254, *P* < 0.01).

The analysis revealed a significant negative correlation between the temperature of the country of origin and the body weight of dog breeds, suggesting that Bergmann’s rule applies even to dogs that have been subjected to strong artificial selection for various purposes over a long period. However, this correlation should be considered as a reflection of the body size characteristics of ancestral indigenous dogs used to produce modern domestic breeds, rather than ongoing adaptations related to heat dissipation efficiency in modern breeds.

Domestic dogs have the greatest body size diversity among all land mammals. Each breed has a unique phenotype that has been subjected to selection pressure by humans according to standards such as those defined by the Fédération Cynologique Internationale (FCI; fci.be/nomenclature). This pressure reduces phenotypic and genetic diversity within breeds but increases diversity between them. However, it is unclear whether environmental factors, such as those referred to in Bergmann's rule, are associated with variations in the genes that determine body size and whether such body size-related genetic variations also affect dog temperament.

Consistent with previous studies, this study found that lightweight dog breeds were more fearful and excitable (McGreevy et al. 2013). In small dogs, undesirable behaviors may be underestimated, whereas in large dogs, such behaviors may be considered more dangerous and eliminated. However, it is possible that genes related to miniaturization are also associated with increased fear and excitability, because these temperaments are commonly found in small dogs, even though some small dogs were miniaturized from larger standard dogs (see Supplementary Discussion).

Contrary to our initial hypothesis, higher temperatures were associated with higher levels of aggression and fear in dogs toward unfamiliar humans and dogs. Our hypothesis was based on Wei et al. (2017), who found that humans are more extraverted and open-minded in warmer environments within physiologically comfortable temperature ranges. However, at uncomfortably high temperatures, increased negative affect may emerge, aligning with the “heat-facilitates-aggression view” proposed in human social science studies. Anderson et al. (1995) suggested that under such conditions, individuals experience greater anger and hostility, which may be misattributed to social interactions, resulting in heightened aggression. Several studies related to this view have shown that aggression decreases when subjects are faced with environmentally and socially more negative or highly repulsive situations under high-temperature conditions (e.g., Palamarek and Gail Rule 1979). These results are not in conflict with the heat-facilitates-aggression view. In order to free themselves from these “bad” situations and reduce overall negative emotions, subjects are thought to switch their response from “fight” to “flight”. Although there may be significant breed differences in the comfort temperature of dogs, it is thought that dogs would generally prefer a lower temperature than is comfortable for humans because their average body temperature is ∼1–2 °C higher than that of humans and because their bodies are covered with fur. It is possible that the heat hypothesis applies to dogs as well and that dogs exposed to the stress of high temperatures become more aggressive and fearful. Paradoxically, it could also be that the lower the temperature, the lower the aggression and fear of dogs. The availability of food and other resources is generally lower in colder climates than in warmer climates. One perspective implies that dogs, which are less aggressive, fearful, and more tolerant of humans, have survived in colder regions by receiving resources from humans. Previous analyses have shown that ambient temperature affects dog-human mutual utility and personhood of dogs (Chambers et al. 2020). A further consideration is the tendency for dogs to be less restrained by their owners in tropical and subtropical latitudes than they are typically in more northern latitudes. Such dogs often tend to live outside the home and may therefore receive less human contact and socialization.

Further discussion is needed to understand the finding that dogs in higher temperature countries exhibit more attachment- and attention-seeking behaviors. Some studies have focused on the relationship between contact behavior and temperature, which is essential for attachment formation, and have reported increased mother–infant contact and group huddling in primates to maintain body temperature in colder environments (Brent et al. 2003). These results seem to contradict the results of the present study. However, direct comparisons with results from primate studies are not appropriate because attachment behaviors observed by C-BARQ are defined as simply being closer to the owner or seeking the owner’s attention by interfering with the attention given to other dogs or humans rather than contact to maintain body heat. Dogs' attachment and attention-seeking behavior toward humans hold significant implications for human–dog interaction and should be carefully considered in light of the migration and lifestyle history of human populations.

Animals quickly adapt their behavior to environmental shifts in a plastic manner, preventing rapid population decline and providing time for genetic change. Initially, behavioral changes in dogs in response to temperature may have been temporary. However, through environmental adaptation and artificial selection, they have become characteristic genetic traits detectable in each breed. Genetic adaptations are more likely to occur in species with shorter generation times, greater genetic diversity, and larger populations. Dogs do not appear to align closely with these conditions. However, modern domestic dogs are very diverse, with 700–800 recognized breeds (356 of which are registered with the FCI; https://www.fci.be/en/), each with a very different morphology and temperament. The diversification of dogs is undoubtedly a result of their coexistence with humans. The changes in dog temperament discussed thus far are also the result of direct or indirect artificial selection and have persisted to the present day. It is possible that the seemingly maladaptive high levels of aggression and fear have also worked as an advantage for humans to use dogs as hunting/gathering partners or guards to watch over crops and remain in present domestic dogs as breed lineage-specific traits.

Overall, temperature was more significantly correlated with temperament items than precipitation, which is consistent with previous studies on human personality traits (Wei et al. 2017). This may indicate that human personality traits influenced by temperature influence the formation of temperament in dogs living in close proximity to humans. Alternatively, it is possible that a similar mechanism exists for the influence of temperature on both human personality traits and dog temperaments.

## Supplementary Material

zoaf044_Supplementary_Data
