# Supplementary material for: Dog temperament is correlated with body weight and climate in country of origin
Source: Curr Zool. 2025 Aug 1;71(6):815–8. doi: 10.1093/cz/zoaf044 (PMC12742383; doi:10.1093/cz/zoaf044)
Supplement: zoaf044_Supplementary_Data [file zoaf044_supplementary_data.zip › A Supplementary Material.docx]

Dog temperament is correlated with body weight

and the climate of origin

Ai Matsumoto¹, James A. Serpell², Miho Nagasawa¹, and Takefumi Kikusui¹*

1 School of Veterinary Medicine, Azabu University

2 School of Veterinary Medicine, University of Pennsylvania

1-17-71 Fuchinobe, Chuo-ku, Sagamihara, Kanagawa 252-5201, JAPAN

Tel/Fax +81-042-769-1853

*Correspondence

Takefumi Kikusui, PhD, DVM

School of Veterinary Medicine, Azabu University

1-17-71 Fuchinobe, Chuo-ku, Sagamihara, Kanagawa 252-5201, JAPAN

Tel/Fax +81-042-769-1853

Email: kikusui@azabu-u.ac.jp

Keywords

breed, temperature, aggression, physiological parameter

Supplemental Results

Correlation analysis

Forest plots of the correlation between the C -BARQ factor and temperature, precipitation, and weight are shown below (Figure S1).


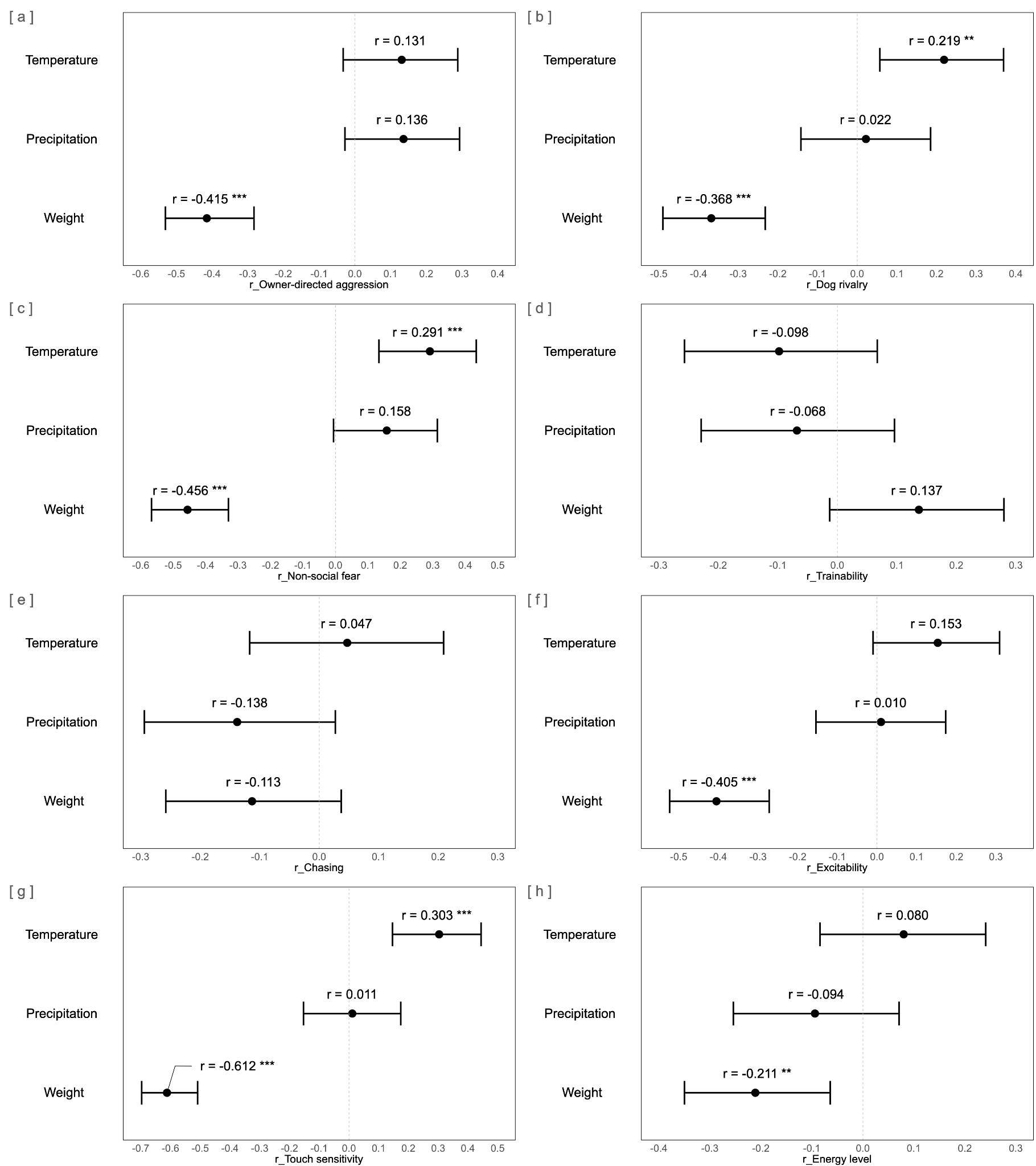


Figure S1. Forest plots showing the correlations between the average annual temperature, annual precipitation, breed-average body weight, and behavioral trait scores from the C-BARQ. Panels A–F correspond to different behavioral traits: [a] Owner-directed aggression, [b] Dog rivalry, [c] Non-social fear, [d] Trainability, [e] chasing, [f] Excitability, [g] Touch sensitivity, and [h] Energy level. Dots indicate Pearson's correlation coefficients (r), and horizontal bars represent the 95% confidence intervals. Asterisks denote statistical significance (*p < 0.05, **p < 0.01, ***p < 0.001).

Validation of demographic variables

Fisher’s exact tests indicated no significant differences in sex ratio among breeds (all p > 0.05, [Table S2](https://docs.google.com/spreadsheets/d/16eQVHgmogdGdIR0ukzSJ4cjcVkSNqGRN/edit?gid=1644513009#gid=1644513009&range=A1)).

Kruskal-Wallis test revealed significant differences in age distributions among breeds (H=2196.440, df=172, p < 0.001, [Figure S2](https://drive.google.com/file/d/1IQNww3jUnfFZVRRk4JbsyLJMLlkDC-K4/view?usp=drive_link)). Although variation in age distribution was observed, age was not statistically controlled in the subsequent analyses, given the exploratory nature of this study and the primary focus on historical environmental factors shaping breed-specific temperament traits. Nevertheless, the potential influence of age distribution should be taken into account when interpreting the findings.

(Due to the large file size, Figure S2 is provided separately as a standalone file in the supplementary materials.)

Figure S2. Age distribution histograms for each breed included in the study. The x-axis represents the age in years, and the y-axis represents the number of individuals.

Supplemental Methods

Analysis of C-BARQ data

The Canine Behavioral Assessment and Research Questionnaire C-BARQ (Hsu and Serpell, 2003) was used to collect demographic and temperament data on 70,122 pet dogs. Breeds with fewer than 30 samples were excluded to ensure sufficient representation of breed characteristics. Responses were excluded if the dog's age exceeded 20 years or if its weight was over 150 kg, as reported by the owner, due to potential unreliability. Mixed-breed data were also excluded to clarify the genetic impact on temperament.

In the analysis, data from 46,272 dogs (173 breeds, 46.5% female, mean age 3.70 ± 10.61, response period: 3/7/2008 - 2/18/2022) were used.

Scores for 14 categories of behavior assessed in C-BARQ were calculated as the average of the raw response values to the corresponding questions. Each of the 46,284 data points was transformed by adding 1 and applying a logχ+1 transformation to handle zero or near-zero values appropriately. Mean values were then calculated for each breed ([Table S1](https://docs.google.com/spreadsheets/d/16eQVHgmogdGdIR0ukzSJ4cjcVkSNqGRN/edit?gid=1170728377#gid=1170728377&range=A1)).

Country of origin of the dog breed

The country of origin for each dog breed was determined using information from Wikipedia and the American Kennel Club ([Table S1](https://docs.google.com/spreadsheets/d/16eQVHgmogdGdIR0ukzSJ4cjcVkSNqGRN/edit?gid=1170728377#gid=1170728377&range=A1)). In cases where a breed was associated with more than one possible country of origin, the breed was treated as originating from each of those countries. For example, since there is disagreement as to whether the poodle originated in Germany or France, the data were analyzed both as German dogs and as French dogs.

Climate data

Annual average temperature data and annual average precipitation data by country for the period 1961-1999 were obtained from the World Bank's Climate Change Knowledge Portal: Historical Data ([Table S1](https://docs.google.com/spreadsheets/d/16eQVHgmogdGdIR0ukzSJ4cjcVkSNqGRN/edit?gid=1170728377#gid=1170728377&range=A1)). Data for the U.S. and China were not used in the analysis because of the large sizes of these countries and the large geographical differences within each country, which may render the use of averages by country inappropriate.

Statistics analysis

- Correlation analysis

To examine the relationships between breed-specific average C-BARQ scores and temperature, precipitation, and breed-average body weight, correlation analyses were conducted using the statistical software JASP version 0.19.3 (University of Amsterdam, Netherlands, https://jasp-stats.org/). Pearson’s correlation coefficients were calculated, and the significance level was set at α = 0.05.

- Validation of sex ratio and age distribution

To assess potential demographic biases across breeds, we analyzed differences in sex ratio and age distribution. Sex ratio differences were examined using Fisher's exact tests for each breed comparison, conducted in R version 4.5.0.

Age distribution differences were assessed using Kruskal-Wallis test in JASP version 0.19.3.

Supplemental Discussions

Association between miniaturization genes and aggression/fear

Zapata et al. found that variation at loci associated with aggression and fearfulness were also associated with breed miniaturization (Zapata I et al. 2016). For example, the Chr15 IGF1 gene, which contributes the most to miniaturization across breeds, was also associated with separation anxiety, touch sensitivity, aggression toward owners, and dog rivalry. However, it remains unclear what determines the allele frequencies at these loci that are associated with both dog size and temperament, as well as which traits were the primary factors for selection. In the current study, temperature was found to be associated with both canine temperament and body size, suggesting that temperature may have influenced allele frequencies, which in turn could have impacted temperament. Future studies examining the relationship between allele frequencies of miniaturization-related genes and aggression/fear-related genes, as well as temperature, may help clarify this. Additionally, behavioral experiments involving mice that incorporate these genes could further elucidate these interactions.

References

[Zapata I, Serpell JA, Alvarez CE. Genetic mapping of canine fear and aggression. BMC Genomics. 2016 Aug 8;17(1):572.](https://www.zotero.org/google-docs/?EkXfeB)
